# Perception of agricultural drought resilience in South Africa: A case of smallholder livestock farmers

**DOI:** 10.4102/jamba.v13i1.984

**Published:** 2021-06-22

**Authors:** Yonas T. Bahta

**Affiliations:** 1 Department of Agricultural Economics, Faculty of Natural and Agricultural Sciences, University of the Free State, Bloemfontein, South Africa

**Keywords:** agricultural drought, government, resilience, smallholder livestock farmers, social networks

## Abstract

Worldwide drought has significance and continues to pose long-lasting effects on the agricultural sector, including South Africa. The recurring drought is a major challenge to smallholder livestock farmers in the Northern Cape Province of South Africa. This study assesses the perception of smallholder livestock farmers towards agricultural drought resilience. The study utilised a perception index score using primary data collected from 207 smallholder livestock farmers following a structured questionnaire survey and multistage sampling procedures. The study found that the average perception index of the role of social networks and government to enhance agricultural drought resilience was negative, which implied that their role in enhancing resilience towards agricultural drought was insufficient. However, the perception of smallholder livestock farmers on the role of social networks was lower than the role of government. This study recommends coordination and cooperation amongst all role players to reinforce strategies to enhance smallholder livestock farmers’ resilience. This includes coordinator amongst the local, provincial government, African Farmers’ Association of South Africa, extension officers, private sectors, monitoring agencies in terms of reliable early warning information and communication amongst decision-makers. Collaboration amongst government departments at the national and provincial levels should be strengthening to enhance farmer’s resilience. The collaboration includes the Department of Agriculture, Forestry and Fisheries at the national level, Provincial Departments of Agriculture, National and Provincial Disaster Management Centres, South African Weather Service and Department of Water Affairs. Smallholder livestock farmers’ awareness of the significance of social networking and government participation should be promoted.

## Introduction

Resilience^1^ is an important concept across all disciplines for examining responses to changes in human (including transformability, adaptability and persistence) and ecological systems (Cote & Nightingale 2011; Downes et al. 2013; Folke et al. 2010; Rockenbauch & Sakdapolrak 2017). Resilience can encompass many spheres, including development, residence, climate, community and disaster (Folke et al. 2010). Agricultural drought,^2^ specifically recurrent agricultural drought, is a disaster that deteriorates smallholder farmers’^3^ resilience capabilities in many aspects. Furthermore, agricultural drought has severe implications for smallholder farmers in social, environmental and economic terms.

Smallholder agriculture, in general, and smallholder livestock farmers, in particular, are characterised by small production volumes of variable quality that reflect limited access to inputs, market, information, insurance, infrastructure and government support (such as assistance from extension offices), as a result, affect the resilience of smallholder livestock producers (Bahta, Jordaan & Muyambo 2016; Jordaan 2011; Von Loeper et al. 2016). Extension and advisory services may provide an opportunity for strengthening the resilience of smallholder livestock producers by increasing their access to tangible and intangible resources, such as inputs and information regarding weather and climate change, market prices, regulatory structures, quality standards and consumer demands so that farmers can make informed decisions. For mitigating risk, extension services can link up with various stakeholders, including insurance providers, input dealers and other market players, to enhance the resilience of smallholder farmers (Davis, Babu & Blom et al. 2014). Infrastructure such as fencing, breeding infrastructure, adequate market facilities and transportation system (rail and road) is most important for market development in terms of distribution to enhance the resilience of smallholder livestock farmers (McDermott et al. 2010).

In South Africa, livestock production has great potential to alleviate household food insecurity and poverty (Mapiliyao et al. 2012). The livestock industry contributes approximately 48% of South Africa’s agricultural output, employs approximately 500 000 people nationwide and occupies 53% of agricultural land (Blignaut et al. 2014; Department of Agriculture, Forestry and Fisheries [DAFF] 2016, 2018). According to the president of Agriculture Northern Cape, as cited by Coleman (2017), livestock farmers in the Northern Cape recently lost entire herds and reduced their livestock numbers by more than 30% in the worst drought since 1982. The 2015/2016 year is the worst drought in South Africa, in general, and Northern Cape Province, in particular. The situation was aggravated by insufficient drought relief schemes, inadequate policies as well a lack of disaster measures and collaboration amongst different role players (Mare, Bahta & Niekerk 2018). Resilient livestock farmers can respond, absorb and recover from drought effects. Jones and Thornton (2009) highlighted that building resilience is essential in reducing agricultural production vulnerability to the variability of climate shocks. Consequently, assessing the perception of smallholder livestock farmers towards agricultural drought resilience is vital for the design and development or improvement of agricultural drought resilience strategies.

Existing international studies, such as those by Marshall (2007), address the question of whether policy perception can erode or enhance the resilience of commercial farmers using survey and descriptive statistics and found that a negative perception of policy was found to significantly and adversely influence the behaviour and emotional response of commercial farmers and influences their resilience. Caldwell and Boyd (2009) quantitatively analysed the impact of drought with emphasis on the concept of resilience in times of stress using a survey and revealed that a wide range of coping strategies was being utilised by these families from problem-focussed coping, optimism and positive appraisal to less adaptive strategies such as cognitive dissonance, denial and avoidance of negative social influences. Buikstra et al. (2010) assessed components of community and individual resilience using a participatory approach, and they found that recognising environmental and economic factors, infrastructure and support services, as enhancing resilience. Darnhofer, Fairweather & Moller (2011) assessed the resilience of family farms concerning the role of farm type and ecological dynamics, and they found that how resilience theory applied to farming may provide a more comprehensive route to achieving sustainability and offers rules of thumb as guides to building farm resilience. Jacobi et al. (2015) assessed agroecosystem resilience quantitatively and found that it enhances the social process of farmers’ integration into cooperatives, and their reorientation towards organic principles and diversified agroforestry enhances their resilience. Darnhofer et al. (2016) assessed the resilience of family farms, that is, the ability to persist over the long-term through buffering shocks and adapting to change. They found that a relational approach would thus contribute to overcoming a one-sided focus on states and stability, shifting attention to the patterns of relations that enable transformational change.

In the African context, studies, such as those by Hudson (2002); Shewamake (2008); Slegers (2008); Gandure, Walker and Botha (2013) and others concentrated on preparedness, impact on and response by the farming community to drought, perceptions on climate change, the inter-relationship between land degradation and drought, rainfall and drought, scarcity of water and coping responses. There are no many studies on the perception of agricultural drought resilience. Bahta et al. (2016) assessed crop and livestock communal farmers’ perception of agricultural drought; application of resilience theory to farming with an insight into drought vulnerability to their farming operations, gender, social network, the role of government, stress and security and safety in the Eastern Cape province of South Africa and found out that perceptions held by communal farmers indicate that they receive inadequate government support; they do not consider social networks as being effectively involved in drought risk reduction, gender stereotyping and psychological stress and experience high levels of stock theft and insecurity in their farming.

However, no study assessed agricultural drought resilience and smallholder livestock farmers’ perception of agricultural drought resilience in relation to social networks and households. Therefore, this study attempts to fill this gap in the literature and knowledge with respect to agricultural drought resilience, social networks and participation of the government. The study’s main aim was to assess the perception of smallholder livestock farmers towards agricultural drought resilience. The findings of this study will help policymakers formulate appropriate policy interventions that boost smallholder livestock farmer’s resilience to agricultural drought.

## Literature review

Natural disasters such as drought constitute direct and indirect threats to the livelihoods and food security of smallholder farmers in the world (FAO 2017). Often, the effects of drought gather gradually over a certain period and can remain for quite a long time after it has departed; it is difficult to determine when the drought started and ended (Wilhite 2000). Climate change exposes rural households and farmers to new and unfamiliar circumstances (Osbahr et al. 2008). Globally, livestock production provides food and livelihood to approximately one billion poor people, mostly in dry and infertile regions where other agricultural practices are less practicable (Rojas-Downing et al. 2017). Different barriers and motivators influence livelihood responses, which comprise gender, social norms, ethnic groups, household assets, individual perceptions, class and networks (Osbahr et al. 2008).

In South Africa, drought has a major impact on livestock production. Drought leads to the reduction of natural grazing (grass) and water. Over the years, the livestock numbers have been increasing. However, the livestock numbers declined slightly in 2016. Livestock numbers declined by 1, 21% compound annual growth rate (CAGR) from 44, 4 million livestock numbers in 2012 to 42, 3 million livestock numbers in 2016. The decline in the number of livestock in South Africa could be attributed to the severe drought, amongst others, which left most farmers – especially smallholder farmers – vulnerable (Matlou & Bahta 2019).

Droughts may be identical in terms of their intensity, duration and spatial characteristics for a specific region or area, but the effects will not be the same. According to Dellal and McCarl (2010), the effects of drought are based on the frequency, severity, degree and vulnerability of the region or area. The effect of drought can be seen from environmental, economic, social and food security aspects.

The development of resilient agricultural systems is essential because many individuals, communities or societies rely upon the provisioning of ecosystem services such as fodder, food and fuel for their livelihoods (Lin 2011). To manage and enhance resilience, individuals, communities and organisations need to anticipate and prepare for each climate-related challenge (Marshall 2010). Resilience presents a new and valuable context of analysis and perception on how the environment, communities, organisations and individuals can adjust in a changing world facing several uncertainties and difficulties (Folke et al. 2016).

To enhance smallholder farmers’ resilience through social network and government coordination of agricultural drought resilience, collaboration, community involvement, be a member of cooperatives, assistance from relatives (family members) and assistance from neighbours is crucial. Social networks can be either formal (e.g. the farmers’ associations such as African farmers Association of South Africa and drought mitigation clubs) or informal (e.g. church groups, women’s groups, stokvels, burial societies, extended family networks and neighbourhood groups) (Wongbusarakum & Loper 2011). They meet and train each other on agricultural drought resilience and mitigation strategies and support each other when drought occurs. Members of social networks share mutual assistance and support when the need arises, such as providing farming knowledge and food in inadequate food supplies. They can call on each other for help and have rights and access to some resources because of their group membership status (Hassen 2008). Iglesias, Moneo and Quiroga (2007) established that when farmers participate in local institutions, their resilience to agricultural drought enhances significantly reduced. Their involvement in planning and other activities influences the social networks in such a way that they will develop social capital to strengthen their resilience.

Smallholder livestock farmers with a strong social network system cope better with drought than those without any or those with weak and ineffective social networks (Stone 2000). Hassen (2008) indicated that members of the social network would be able to help each other and access resources. A robust institutional background is essential for the promotion of resilience in the face of hazardous events. Information could then be disseminated easily to the public, ensuring the facilitation of emergency preparedness, pre-disaster planning and enhance resilience of smallholder livestock farmers (Vincent 2004).

## Methodology

### Study area

**FIGURE 1:**
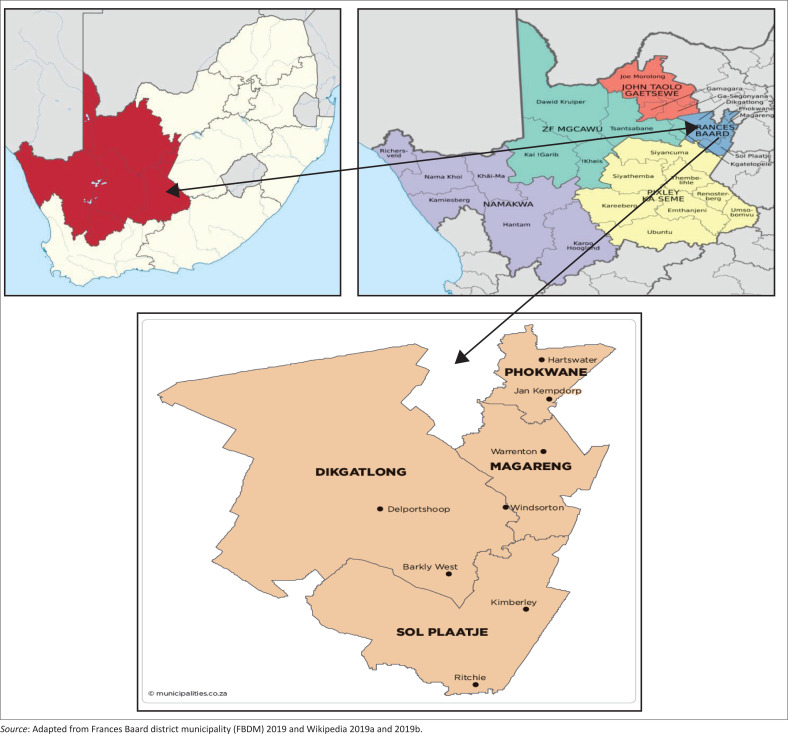
A map of South Africa highlighted the Northern Cape Province, district municipalities of Northern Cape and the four local municipalities of Frances Baard District Municipality Map.

The Northern Cape Province is the largest province of South Africa, comprising 36 million hectares (29.5%) of South Africa’s total land area (Bapela & Mariaba 2002; Dludla 2014). The study focussed on the France Baard District Municipality (FBDM), consisting of four local district municipalities, namely Dikgatlong, Magareng, Phokwane and Sol Plaatjie (Figure 1). According to the census of 2011, the FBDM has a population of 382 087 with 95 931 households, which accounted for 31.8% of the Northern Cape households with 3.98 people per household. The local municipality of Dikgatlong, Magareng, Phokwane and Sol Plaatje had a population size of 46 842, 24 203, 63 000 and 248 042, respectively (France Baard District Municipality [FBDM] 2016).

### Sampling procedure

A multiple-stage sampling procedure was employed. Firstly, the Northern Cape Province was chosen from the nine provinces of South Africa. According to Statistics South Africa (Stats SA 2016), approximately 75% of agricultural households in 2016 were involved in livestock production, compared to mixed farming (10%) and crop only (15%) in the Northern Cape. In 2016, Eastern Cape (65%), Western Cape (18%), Free State (26%), KwaZulu-Natal (48%), North West (47%), Gauteng (10%), Mpumalanga (34%) and Limpopo (39%) of the agricultural households engaged in the livestock production. With other South African provinces, the province was declared a disaster zone in 2017/2018 by the South African government because of agricultural drought. Secondly, four district municipalities in the province were randomly selected using balloting and included Dikgatlong, Magareng, Sol Plaatjie and Phokwane.

Appropriate sample sizes were calculated for continuous and categorical data (Cochran 1997). The questionnaire included both continuous and categorical data, which comprised socio-economics characteristics, the role of a social network, the role of government and drought vulnerability. Thus, to ensure that the sample size was appropriate, a simple random sampling formula was applied (Bartlett, Kotrlik & Higgins 2001; Cochran 1997). Based on the formula, 207 smallholder livestock farmers were selected from 868 farmers registered to receive drought relief from government in the Northern Cape Province of South Africa for face-to-face interviews conducted from July to September 2018 using a structured questionnaire (Northern Cape Department of Agriculture, Forestry and Fisheries 2018). Some of the questionnaires (role of government and social network) are available in Appendix 1).

### Data analysis

The data were analysed using the perception index score for agricultural drought resilience. A perception index score is a composite index that ranks social indicators such as the role of social networks and government, based on how the smallholder livestock farmers perceive its influence towards the resilience of agricultural drought (Transparency International 2014). When there is a lack of data to do a detailed analysis of natural hazards such as agricultural drought, a perception index score becomes very important for such analyses (Dwyer et al. 2004). Bahta et al. (2016) applied the perception index for communal farmer’s perception of drought in the Eastern Cape province of South Africa. The role of government and social networks perceived by smallholder livestock farmers’ to build agricultural drought resilience was of binary nature of the response and a respondent’s choice lies on ‘agree’ and ‘disagree’ (with the statements indicated in Appendix 1), with numbers of respondents and the ‘agreed’ respondents assigned a positive value (+1) and ‘disagree’ respondent assigned a negative value (−1). A perception index score expressed as the mean score:

$$\text{Mean score}=\frac{Number\;of\;Agree\;respondents-Number\;of\;Disagree\;respondents}{Total\;number\;of\;respondent}$$ [Eqn 1]

The closer the mean score was to +1, the greater the positive perception, and the closer the mean score was to −1, the greater the negative perception. Udmale et al. (2014) and Bahta et al. (2016) highlighted that in mixed farming variables considered to analyses perception of farmers to agricultural droughts such as the role of gender, socio-economic activities, drought adaption, drought mitigation measure, psychological stress, level of drought risk to farmers farming operation, security and safety threats on their farm and other indicators. The indicator variables included in this study were the role of government and social networks. In this study, the selection of variables involved deductive approaches (Adger et al. 2004). The deductive approaches deal with theoretical relationships and the suitability of assigning values and weights. The variables selected were based on relevance, availability and ease of understanding and collecting information. The variables selected were not solely based on literature but also on the experience of the researchers in the study area.

## Results and discussion

### Socio-economic characteristics of smallholder livestock farmers

A summary of the socio-economic characteristics of the respondents is shown (Table 1). The average age of the respondents was 55 years (median 56 years). This finding aligned with that of Badenhorst (2014) who found that the majority of farmers were not young. This implied that the younger generation did not consider agriculture as a profession. The highest proportions of the respondents were married (67%), single (20%), widowed (11%) or divorced (2%). Most (97%) of the respondents had one to five members in their household, and most were men (81%). This could have implied that a gender stereotype still existed in farming from the sampled respondents. Almost half (48%) of the respondents had attended high school, and 5% and 4%, respectively, possessed a diploma and degree. To develop a household’s agricultural drought resilience, education plays an important role. The average farming experience was 12 years. Respondents farmed with sheep (25%), goats (30%) and cattle (45%).

**TABLE 1:** Socio-economic characteristics of respondents (*n* = 207).

Characteristics	Mean	Median	Minimum	Maximum	Sub-characteristics	Frequency	%
Age (years)	55	56	21	89	18–40	33	16
					40–65	120	58
					> 65	54	26
Marital status	-	-	-	-	Single	41	20
					Married	138	67
					Widowed	24	11
					Divorced	4	2
Number of household members	2	1	1	11	1–5	201	97
					5–10	4	2
					> 10	2	1
Gender	-	-	-	-	Male	168	81
					Female	39	19
Education level	8	8	0	16	No schooling	23	11
					Elementary	65	32
					High school	100	48
					Diploma	10	5
					Degree	9	4
Farming experience (years)	12	10	1	40	1–10	119	58
					10–20	63	30
					> 20	25	12
Number of livestock	-	-	-	-	Cattle	4300	45
					Sheep	2425	25
					Goats	2928	30

### Respondents’ perceptions on level of drought vulnerability

Almost two-thirds (64.25%) of respondents perceived that their farming operations were very highly vulnerable to agricultural drought, 16.43% highly vulnerable and 13.53% moderately vulnerable. Finally, yet importantly, 1.93% and 3.86% of respondents perceived themselves as having zero and low vulnerability to agricultural drought, respectively. Amir Faisal, Polthanee and Promkhambut (2014) highlighted that farmers are aware of the intensity and the nature of recurrent drought. The experience and the severity of drought ranged from 2 to 5 years on respondents’ farms. The most severe years of drought which respondents prioritised were 2015–2016, 2016–2017; 1982–1983, 1992–1993, 2009–2010 and 2012–2013.

### Respondents’ perceptions on the role of social networks

Social networks play an essential role in agricultural drought resilience. Many aspects that positively correlate with social networks include the human ability to absorb, buffer and initiate social innovations, to act collectively and the capacity to change and adapt strategies (Adger 2003; Matin et al. 2015; Moore & Westley 2011; Newman & Dale 2005; Tobin et al. 2014). Social networks can be either informal (e.g. neighbourhood groups, extended family networks, burial societies, women’s groups and church groups) or formal (e.g. drought mitigation clubs, cooperatives and farmers’ associations) (Bahta et al. 2016; Wongbusarakum & Loper 2011).

Respondents’ perceptions of the role of social networks in enhancing resilience are presented (Table 2). Social networks are divided into six indicators. Firstly, coordination and included institutions (e.g. farmers’ organisations, cooperatives, Non-Governmental Organisations [NGOs], church clubs and family networks) that had the ability to coordinate activities of agricultural drought resilience. Secondly, collaboration, including the community’s ability to collaborate with existing institutions and groups in enhancing agricultural drought resilience. Thirdly, involvement, namely the efforts made by the communities in enhancing resilience. Fourthly, cooperatives, which represented an association established to help with resources to cope with agricultural drought or resilience. Fifthly, relatives, which entailed family members helping during recurrent drought to reduce the burden of the shock. Sixthly, neighbours who included the people who lived around the farm and assisted with tending livestock.

**TABLE 2:** Respondents’ perception on social network involvement in agricultural drought resilience (*n* = 207).

Variable	Agree (+1)	Agree (%)	Disagree (−1)	Disagree (%)	Mean score = (agree–disagree/*n*)
Coordination	20	9.66	187	90.34	−0.81
Collaboration	58	28.02	149	71.98	−0.44
Involvement	59	28.50	148	71.50	−0.43
Cooperatives	58	28.02	149	71.98	−0.44
Relatives	37	17.87	170	82.13	−0.64
Neighbours	57	27.54	150	72.46	−0.45
Average	-	23.27	-	76.73	-
Total mean score	-	-	-	-	−3.21
Perception index score	-	-	-	-	−0.54

The perception index for the social networks is −0.54 (Table 2). The respondents do not consider social networks efficient in enhancing resilience towards agricultural drought. The respondents’ strongest negative perception (−0.81) is for coordination. Even though negative, community involvement in resilience activities is the strongest aspect of other social network indicators. About 9.66% of the respondents perceived coordination as a positive indicator (Table 3); this could have been because of the absence of effective and efficient farmers’ associations. Only 28.5% of the respondents felt that they are involved in agricultural drought resilience. It is inferred that social networks (23.27%) did not play an important role in enhancing agricultural drought resilience. However, literature shows that social networks are very important in the reduction of social vulnerability (Kuhlicke et al. 2011; Muyambo, Jordaan & Bahta 2017).

**TABLE 3:** Respondents’ perception of government’s role in agricultural drought resilience (*n* = 207).

Variable	Agree (+1)	Agree (%)	Disagree (−1)	Disagree (%)	Mean score = (agree–disagree/*n*)
Previous help	125	60.39	82	39.61	0.21
Government assistance	140	67.63	67	32.37	0.35
Government interest	124	59.90	83	40.10	0.20
Government training	105	50.72	102	49.28	0.01
Government polices	87	42.03	120	57.97	−0.16
Government farming practice	49	23.67	158	76.33	−0.53
Average	-	50.72	-	49.28	-
Total mean score	-	-	-	-	0.08
Perception index score	-	-	-	-	0.013

### Respondents’ perception on the role of government

A solid and functional institution is significant for enhancing agricultural drought resilience by disseminating information and policies to the public to ensure proper preparedness and planning (Vincent 2004). Table 3 presents respondents’ perceptions of the government’s role in enhancing resilience, and six indicators were used. Firstly, previous help, the government’s past participation in agricultural drought resilience. Secondly, government assistance in supplying resources needed to build resilience (e.g. finances for farm input and fodder). Thirdly, government interest in agricultural drought resilience issues. Fourthly, government training, where the government participated in the training, dissemination of information and livestock management. Fifthly, government policies with the dissemination of national or regional drought policies. Sixthly, government farming practice with support to improve.

A positive perception index of 0.013 (Table 3) implied that respondents perceived that the government-supported them to build their resilience; however, respondents indicated that the government needed to do more to support them. They elaborated that support from the government was often not on time and not good enough to enhance respondents’ productivity to full capacity and enhance their resilience towards agricultural drought. As indicated (Table 3), 140 (67.63%) respondents received assistance from the government in the form of farm input, finance and food. Most (62.63%) respondents claimed that they received assistance from the government for farm input, including fodder, 1% received financial assistance and the rest (4%) received food assistance during agricultural drought. Jordaan (2011) emphasised that in the Northern Cape Province of South Africa, there was inadequate drought support, late delivery of drought support and institutions incapable of service delivery.

Half (50.72%) of the respondents explained that the government provided training related to livestock management. Less (42.03%) respondents gained access to information related to policies on agricultural drought resilience. This was confirmed by an extension officer, who stated that they did not train or provide information about agricultural drought resilience and vulnerability.

### Comparison of indicator variables

The average perception index score on the role of the social networks was 23.27%, whilst that of the role of government was 50.72%. This result implied that respondents perceived that government and social networks impacted building resilience towards agricultural drought. The percentage proportion of respondents’ perception of the role of social networks had a lower impact in building resilience than the role of government. Overall, the average perception index of the role of social networks and government was –0.26 (–0.54+0.013/2) (Table 2 and Table 3), indicating that their role in enhancing resilience towards agricultural drought was insufficient. Opiyo, Wasonga and Nyangito (2014) highlighted that social networks, social support and government support strengthen the resilience of households and farmers.

### Correlation analysis of indicator variables

Perception of respondents on the role of the social networks indicator’s involvement was positive and significantly correlated with collaboration at 1%, respectively (Table 4 and Table 5). Engagement in cooperatives was positive and significantly correlated with collaboration and involvement at 1% level. This implied that the more farmers are engaging with cooperatives, the more reducing the burden of agricultural drought.

**TABLE 4:** Pearson’s correlation estimate for the indicator variable: Social network role.

Variable	Social network role										
	Coordination		Collaboration		Involvement		Cooperatives		Relatives		Neighbours
	Correlation	*p*-value	Correlation	*p*-value	Correlation	*p*-value	Correlation	*p*-value	Correlation	*p*-value	
Coordination	1	-	-	-	-	-	-	-	-	-	-
Collaboration	0.014	0.8366	1	-	-	-	-	-	-	-	-
Involvement	0.011	0.8767	0.845*	0.0000	1	-	-	-	-	-	-
Cooperatives	−0.025	0.7229	0.337*	0.0000	0.305*	0.0000	1	-	-	-	-
Relatives	0.061	0.3840	0.018	0.7994	0.096	0.1667	0.040	0.5702	1	-	-
Neighbours	0.128	0.0664	0.555*	0.0000	0.569*	0.0000	0.322*	0.0000	0.108	0.1228	1

*, **, *** Correlation is significant at the 0.01, 0.05 and 0.1 (2-tailed).

**TABLE 5:** Pearson’s correlation estimate for the indicator variable: Government role.

Variable	Government role										
	Previous help		Government assistance		Government interest		Government training		Government policies		Government farming practice
	Correlation	*p*-value	Correlation	*p*-value	Correlation	*p*-value	Correlation	*p*-value	Correlation	*p*-value	
Previous help	1	-	-	-	-	-	-	-	-	-	-
Government assistance	0.609*	0.0000	1		-	-	-	-	-	-	-
Government interest	0.668*	0.0000	0.602*	0.0000	1		-	-	-	-	-
Government training	0.545*	0.0000	0.502*	0.0000	0.692*	0.0000	1		-	-	-
Government policies	0.537*	0.0000	0.429*	0.0000	0.564*	0.0000	0.730*	0.0000	1		-
Government farming practice	0.242*	0.0067	0.195***	0.0728	0.201**	0.0565	0.208**	0.0397	0.257*	0.0028	1

*, **, *** Correlation is significant at the 0.01, 0.05 and 0.1 (2-tailed).

Assistance from neighbours was positive and significantly correlated with collaboration, involvement and cooperatives at 1% level (Table 4 and Table 5). This implies that communication and interaction between respondents and institutions should be improved in order to enhance the resilience of respondents.

Respondents’ perceptions on the role of government are presented (Table 4 and Table 5). Most indicator variables under government role in enhancing agricultural drought resilience were significantly correlated with each other at 1% levels and positive. For example, government assistance, which provided resources to farmers to enhance resilience in the form of finance, farm inputs and fodder, was positive and significantly correlated with the government’s previous involvement in agricultural drought resilience. This implied that the government’s participation in the dissemination of information including policies, training related to agricultural drought resilience and access to resources including funding should be increased through vigorous means such as extensive extension officer, easily accessed notice boards, popular media (local magazines) and aggressive media involvement, which are accessible to smallholder livestock farmers.

### Correlation analysis between social network and the government

To assess the correlation between social networks and government indicators, the principal component analysis is utilised by generating a single component or index of government and social networks, and then, tested the correlation between the government and social network indicators.

The correlation between social networks and government established almost all of them at 1% level significant (Table 6). Social network variable coordination was positive and significantly correlated with government variable indicators of government past help, government interest, government policies and government farming practice at 1%, respectively (Table 6). This implies that to achieve the government involvement, such as involvement in agricultural drought resilience in the past, government interest in agricultural drought resilience and impacts in the community, government dissemination of national or regional drought resilience policies and government supplying of resources to cope with agricultural drought, coordination of different stakeholder is necessary.

**TABLE 6:** Correlation estimate between government and social network roles.

Social network role variable	Government role											
	Previous help		Government assistance		Government interest		Government training		Government policies		Government farming practice	
	Correlation	*p*-value	Correlation	*p*-value	Correlation	*p*-value	Correlation	*p*-value	Correlation	*p*-value	Correlation	*p*-value
Coordination	0.2314*	−0.0008	0.0972	−0.1635	0.2008*	−0.0037	0.1261	−0.0702	0.2149*	−0.0019	0.2026*	−0.0034
Collaboration	0.0874	−0.2102	0.0964	−0.1672	0.1373*	−0.0485	0.2276*	−0.001	0.2686*	−0.0001	0.0322	−0.6456
Involvement	0.0738	−0.2907	0.1021	−0.1431	0.1672*	−0.016	0.2370*	−0.0006	0.3013*	0.000	0.0764	−0.274
Cooperatives	0.2004*	0.000	0.0998	−0.1525	0.1847*	−0.0077	0.1898*	−0.0062	0.2098*	−0.0024	0.1976*	−0.0043
Relatives	0.1201	−0.0849	0.0792	−0.2568	0.0987	−0.1572	0.0311	−0.6568	0.0579	−0.4072	0.0665	−0.3411
Neighbours	0.1013	−0.1465	0.1322	−0.0576	0.063	−0.3671	0.11	−0.1145	0.2137*	−0.002	0.2164*	−0.0017

* , Correlation is significant at the 0.01 (2-tailed).

A social network variable collaboration was positive and significantly correlated with government variable indicators of government interest, government training and government policies at 1%, respectively. This implies that collaboration, including the community’s ability to collaborate with existing institutions and groups in enhancing agricultural drought resilience, needed to government interest in agricultural drought resilience and impacts in the community, government trains the community and government dissemination of national or regional drought resilience policies required.

A positive and significant correlation between social network variable, involvement and government variable government interest, government training and government policies exists at 1%, respectively.

## Conclusion and recommendation

Climatic variability is an unavoidable phenomenon. These uncertainties affect smallholder farmers’ livelihoods when they lose their herds and capital. The study’s main aim was to assess the perception of smallholder livestock farmers towards agricultural drought resilience and gain an understanding of the role of social networks and government on building resilience towards agricultural drought.

The study found that the average perception index of social networks and government to enhance agricultural drought resilience was negative. This implied that their role in enhancing resilience towards agricultural drought was insufficient. However, the proportion of respondents’ perception of the role of social networks was lower than that of government. This indicated that respondents did not consider social networks efficient in enhancing resilience towards drought. With regard to the role of the social networks, the strongest negative perception was for coordination. Even though community involvement was negative, it was the strongest aspect compared to other social network indicators. Thus, social networks did not play a significant role in enhancing agricultural drought resilience. This could be, the information smallholder livestock farmers have about the social network is not good enough. Hence, smallholder livestock farmers’ awareness of the significance of social networking and government participation should be promoted. All respondents perceived that they were either highly vulnerable or moderately vulnerable to drought, which indicated that they were aware of the intensity and the recurrent nature of agricultural drought.

There are implications in finding that the current social networking groups are not operating efficiently towards building the resilience of respondents. Hence, to build resilience, there should be coordination and cooperation amongst all role players to reinforce policies and strategies to build the resilience of smallholder livestock farmers. This includes coordinator amongst the local provincial government, African Farmers’ Association of South Africa, extension officers, private sectors, monitoring agencies in terms of reliable early warning information and communication amongst decision-makers. Collaboration amongst government departments at the national and provincial levels should be strengthening to enhance farmer’s resilience. The collaboration includes the Department of Agriculture, Forestry and Fisheries at the national level, Provincial Departments of Agriculture, National and Provincial Disaster Management Centres, South African Weather Service and Department of Water Affairs. Their awareness of the importance of social networks and government participation could be enhanced. In particular, the government and the Northern Cape Province should improve access to information, access to training related to agricultural drought resilience, affordability of veterinary service, financing, irrigation and land and strengthening investment in a fodder bank.

## Footnotes

1 Resilience is about having the capacity to sustain in the face of shocks and change by buffering shocks, adapting and transforming in response to change (in this study agricultural drought) (Folke 2016).

2 Agricultural drought is a decline in water availability (precipitation) below the optimal level required (IPCC 2012).

3 Smallholder farmers are those farmers in transition between subsistence and commercial farming.

## Appendix 1

**TABLE 1-A1:** What is the social network’s role in agricultural drought resilience or extent of social network involvement in agricultural drought resilience?

Statement	Disagree/no/−1	Agree /yes/+1
Coordination – Do you think institutions (e.g. farmers’ organisations, cooperatives, NGOs, church clubs and family networks) could coordinate activities of agricultural drought resilience?	-	-
Collaboration – Does the community have the ability to collaborate with existing institutions and groups to enhance agricultural drought resilience?	-	-
Involvement – Does the community attempt to make efforts to involve in enhancing resilience?	-	-
Cooperatives – Do the cooperatives (represented an association established) provide resources to cope with agricultural drought or resilience?	-	-
Relatives – Do relatives (entailed family members) help during recurrent drought to reduce the burden of the shock?	-	-
Neighbours – Do neighbours (people who lived around the farm) provide any support with tending livestock during a drought?	-	-

**TABLE 2-A1:** Indicate the role of the government on agricultural drought resilience.

Statement	Disagree/no/−1	Agree /yes/+1
Governments have successfully led us through agricultural drought resilience in the past	-	-
The government provides funding or assistance for agricultural drought resilience purposes	-	-
Government is interested in agricultural drought resilience and impacts in our community	-	-
The government trains the community and gives knowledge and skills to take charge of agricultural drought resilience effectively	-	-
Government informs us of national or regional drought resilience policies or initiatives that may impact our community	-	-
The government provides resources (government farming practice) we need to cope with agricultural drought (e.g. fodder, water, food parcels)	-	-
